# Investigation of the Antioxidant and Hepatoprotective Potential of *Hypericum mysorense*

**DOI:** 10.3390/antiox3030526

**Published:** 2014-08-12

**Authors:** Raghu C. Hariharapura, Ramamurthy Srinivasan, Godavarthi Ashok, Santoshkumar H. Dongre, Hitesh V. Jagani, Pottekkad Vijayan

**Affiliations:** 1 Department of Pharmaceutical Biotechnology, Manipal College of Pharmaceutical Sciences, Manipal University, Manipal 576 201, India; E-Mail: hiteshjagani@gmail.com; 2 School of Pharmacy, International Medical University, Kuala Lumpur 57000, Malaysia; E-Mail: rsriniv@gmail.com; 3 Radiant Research Services Pvt. Ltd., Bangalore 560 050, India; E-Mail: surya.ashok@gmail.com; 4 Department of Chemical Technology, Dr. Babasaheb Ambedkar Marathwada University, Aurangabad 431001, India. E-Mail: santoshdongre@gmail.com; 5 Department of Pharmaceutical Biotechnology, Jagadguru Sri Shivarathreeshwara (JSS) College of Pharmacy, Ooty 643 001, India; E-Mail: vijayanp4@rediffmail.com

**Keywords:** *Hypericum mysorense*, antioxidant, free radical, hepatoprotective activity

## Abstract

**Background:** *Hypericum* is a well-known plant genus in herbal medicine. *Hypericum mysorense* (Family: *Hypericaceae*), a plant belonging to the same genus, is well known in folklore medicine for its varied therapeutic potential. **Objective:** The aim of the present study was to investigate the different parts of the plant for antioxidant and hepatoprotective properties. **Materials and Methods:** The methanol extracts of *Hypericum mysorense* prepared from various parts of the plant were tested *in vitro* for their free radical scavenging activity against ABTS^•^ (diammonium salt), DPPH^•^ (1,1-diphenyl-2-picrylhydrazyl), NO^•^, O_2_^•−^ and ^•^OH radicals, using standard systems of assays. The total antioxidant capacity, total phenolic and total flavonoid content of the extracts were analyzed. Further, the leaf and flowering top extracts were tested for their *in vivo* antioxidant and hepatoprotective activities on Wistar rats using a carbon tetrachloride-induced hepatic injury model. Results: The leaf and flowering top extract showed potent antioxidant activity and also possessed highest total phenolic and flavonoid content. The antioxidant activity and the total phenolic and flavonoid content present in these extracts showed a good correlation. The leaf and flowering top extracts at 200 mg/kg restored aspartate amino transferase (ASAT), alanine amino transferase (ALAT), alkaline phosphatase (ALP), total bilirubin and protein levels significantly in CCl_4_-intoxicated rats. The tested extracts also showed a significant (*p* < 0.001) reduction in 2-thiobarbituric acid reactive substance (TBARS) levels with an increase in SOD and CAT levels. The histopathology of liver did not show any toxicity after the treatment with the extracts. The active extracts were standardized using two marker compounds, hyperoside and rutin, which were isolated from the plant by HPLC. HPLC studies revealed that the maximum concentration of hyperoside and rutin is present in the flowering top extract.

## 1. Introduction

Reactive oxygen species (ROS) and reactive nitrogen species (RNS) generated in our body are quite reactive and harmful to the cells. If generated ROS and RNS are not scavenged, they can damage important molecules, such as proteins, DNA and lipids, which lead to the development of a variety of diseases, including aging, mutagenesis, carcinogenesis, coronary heart disease, diabetes and neuro-degeneration [1,2,3]. There is an increasing interest in natural antioxidants, namely phenols and flavonoids, present in medicinal and dietary plants, as they might help to prevent oxidative damage to critical cellular constituents. Flavonoids represent a large group of plant polyphenols possessing a wide range of biological activities, *viz*., vasoprotective, anti-inflammatory, anti-hepatotoxic and anti-carcinogenic action [4,5,6]. Besides their biological activity, they are also known to scavenge free radicals, such as ROS and RNS, through electron transfer from flavonoids/phenols towards these oxygen radicals [7,8]. Flavonoids can also prevent the formation of highly reactive ^•^OH radicals through the Fenton reaction by forming chelates with metals, like iron and copper [9].

*Hypericum* (Guttiferae) is a large genus of herbaceous plants, which grows widely in temperate regions and is being used in traditional medicine in various parts of the world [10]. In recent years, the antidepressant activity of *Hypericum perforatum* L., known as St. John’s wort, has caused wide-spread interest in the study of *Hypericum* genus [11]. Compounds isolated from this genus have shown antifungal [12], antibacterial [13], antiviral [14] and anticancer [15] properties. Flavonoids isolated from the *Hypericum* genus have been shown to have several biological activities, such as antidepressant [16], nitric oxide synthesis inhibition [17], antiproliferative [18] and antioxidant activity [19].

*Hypericum mysorense* (HM) is an ornamental bush found in the Konkan and Palani hills at a height of 900–1500 meters. *Hypericum mysorense* is well known in folklore medicine for its varied therapeutic potential, including spasmolytic, hypotensive and antibacterial activities [20,21]. Earlier studies in our laboratory have shown the significant antiviral [22], cytotoxic and antitumor [23] properties of this plant. We compared the antioxidant activities of the methanolic leaf extract from different *Hypericum* species, such as *H. mysorense*, *H. perforatum*, *H. japonicum* and *H. patulum*, were compared. HM with the highest phenol content (24.72 mg/g) showed the highest activity, followed by *H. perforatum* [24]. In continuation of our work in the present study, we investigated the *in vitro* and *in vivo* antioxidant potential and hepatoprotective effect of HM and compared its activity among different parts of the plant.

## 2. Materials and Methods

### 2.1. Collection and Identification of Plant Material

HM was collected from, in and around Ooty, a famous hill station in southern India, belonging to the Nilgiris, a district of Tamil Nadu state. The plant was identified and authenticated by Medicinal Plants Survey and Collection Unit, Ooty, Tamil Nadu, India, where a voucher specimen was preserved for further reference (voucher specimen No. 8570).

### 2.2. Preparation of Extracts

The aerial parts, flowering tops, leaves, root and stem were separated and dried in the shade. Each of these was then separately powdered, sieved (No. 20), weighed and extracted with a Soxhlet extractor (Borosil, Mumbai, India), using methanol. The extracts were concentrated to dryness under reduced pressure and controlled temperature. All extracts were preserved in a refrigerator at 4 °C till further use.

### 2.3. Preparation of Test and Standard Solutions

Extracts of the aerial parts (HMA), flowering tops (HMF), leaves (HML), root (HMR) and stem (HMS) and the standard antioxidants (ascorbic acid, rutin, butylated hydroxy anisole and α-tocopherol) were dissolved in dimethyl sulfoxide (DMSO) and used for the *in vitro* antioxidant assays using seven different methods, except the hydrogen peroxide method. For the hydrogen peroxide method (where DMSO interferes with the method), the extracts and standards were dissolved in distilled methanol and used. The stock solutions were appropriately diluted with the respective solvents to obtain lower dilutions.

### 2.4. Chemicals

1,1-Diphenyl-2-picrylhydrazyl (DPPH), 2,2′-azino-bis (3-ethylbenzo-thiazoline-6-sulfonic acid) diammonium salt (ABTS), butylated hydroxy anisole (BHA) *tert*-butyl hydroperoxide, chlorpromazine, guanosine, tryptophan, ascorbic acid, silymarin and hyperoside were obtained from Sigma-Aldrich Co., St. Louis, MO, USA. Rutin and *p*-nitroso dimethyl aniline (*p*-NDA) was from Acros Organics, Morris Plains, NJ, USA. Naphthyl ethylenediamine dihydrochloride (NEDD) was from Roch-Light Ltd., Suffolk, UK. Nitro blue tetrazolium (NBT) was from SD Fine Chemicals Ltd., Mumbai, India, and 2-deoxy-d-ribose was from Hi-Media Laboratories Pvt. Ltd., Mumbai, India. Sodium nitroprusside was from Ranbaxy Laboratories Ltd., Mohali, India. Sodium carboxy methyl cellulose (CMC), sulfanilic acid and α-tocopherol were from Merck (India) Ltd., Delhi, India. Cupric chloride, ferric chloride, ferrous ammonium sulfate and all other chemicals were of analytical or equivalent grade. All chemicals were used without further purification. The water used for the preparation of solutions was purified through a Milli-Q water purification system (Millipore, Billerica, MA, USA; Specific conductivity <0.1 μs·cm^−1^), and the sample solutions were prepared just prior to carrying out the experiments.

### 2.5. Selection and Maintenance of Animals

Healthy male albino rats of the Wister strain (180–220 g) were obtained from the animals house, J.S.S. College of Pharmacy, Ooty, India, and were maintained under standard environmental conditions (22–28 °C, 60%–70% relative humidity 12 h dark/light cycle) and fed with standard rat feed (Amrut Rat Feed, Nav Maharasthra Chakan Oil Mill Ltd., Pune, India) and water *ad libitum*. The experiments were conducted as per the guidelines of Committee for the Purpose of Control and Supervision of Experiments on Animals (CPCSEA), Chennai, India. The protocol was approved by the institutional animal ethics committee (Approval No. JSSCP/IAEC/Ph.D/PH.BIOTECH/01/2005-06).

### 2.6. In Vitro Antioxidant Activity

Methanolic extracts of different parts of HM and different standards were assessed for *in vitro* antioxidant activity on the basis of the radical scavenging effects of the stable ABTS [25] and DPPH [26] free radicals, hydroxyl radical by deoxyribose [27] and the *p*-NDA method [28], hydrogen peroxide [29], lipid peroxide inhibition using the standard 2-thiobarbituric acid (TBA) colorimetric method [30,31], nitric oxide [32,33] and super oxide radical by the alkaline DMSO method [34]. In all these methods, a particular concentration of the extract or standard solution was used, which, after the addition of all the reagents, gave a final concentration of 1000 μg/mL to 0.45 μg/mL. Absorbance was measured against an appropriate reagent blank. A control test was performed without adding extracts or standards. The results were expressed in IC_50_ values (concentration of test or standard required to inhibit 50% of free radicals). Measurement of total antioxidant capacity [35] and the total phenol [36] and flavonol [37] content of the methanolic extracts of different parts of HM were carried out. The phytochemical screening of methanolic extracts of different parts of HM was performed using standard methods [38,39].

### 2.7. In Vivo Antioxidant and Hepatoprotective Studies

Carbon tetrachloride (CCl_4_)-induced hepatic injury is the model used for *in vivo* antioxidant and hepatoprotective drug screening [40]. The principle causes of CCl_4_-induced hepatic damage are free radical-mediated lipid peroxidation, leading to the disruption of the biomembrane, dysfunction of cells and tissues, decreased levels of antioxidant enzymes and the generation of free radicals [41]. The antioxidant activity involving the inhibition of the generation of free radicals is important in providing protection against hepatic damage.

#### 2.7.1. Preparation of Extracts and Standard

One hundred and 200 mg/mL of HMF and HML were prepared in 0.5% sodium CMC. One hundred milligrams per milliliter of standard silymarin were prepared in 0.5% sodium CMC.

#### 2.7.2. Experimental Design

The animals were divided into seven groups with six animals in each group. Group I served as the normal control, and Group II served as the toxicant control. Both Groups I and II received 1 mL of 0.5% sodium CMC. Group III received standard silymarin at a dose of 100 mg/kg p.o. Groups IV and V received HMF at a dose of 100 and 200 mg/kg p.o. body weight, respectively. Group VI and VII animals received HML at a dose of 100 and 200 mg/kg p.o. body weight, respectively. Silymarin and extracts were administered orally for 7 days. On the eighth day, all groups received 1 mL/kg body weight of CCl_4_, intraperitoneally, except Group I. On the ninth day, the rats were anesthetized using anesthetic ether, and blood was collected from retro-orbital plexus. After collection, the blood was kept at 37 °C for 30 min. Later, it was centrifuged at 2500 rpm for 10 min to separate serum, which was used for biochemical estimations. Later, all of the animals were sacrificed by decapitation. The liver was removed, weighed and homogenized immediately with Elvenjan homogenizer fitted with a Teflon plunger, in ice-chilled 10% KCl solution (10 mg/g of tissue). The suspension was centrifuged at 2000 rpm at 4 °C for 10 min, and the clear supernatant was used for the biochemical estimations. The levels of antioxidant enzymes, *viz*., catalase (CAT), superoxide dismutase (SOD) and lipid peroxidation (LPO or 2-thiobarbituric acid reactive substances (TBARS)) were measured. Marker enzymes, such as alanine amino transferase (ALAT), aspartate amino transferase (ASAT), alkaline phosphatase (ALP), total bilirubin (TB) and total protein (TP) were measured spectrophotometrically in serum and liver samples using commercially available Ecoline diagnostic kits (Merck, Mumbai, India) [42,43].

### 2.8. Histopathological Studies

On the ninth day of the experiment, all the animals were sacrificed by decapitation, and the liver was dissected out, the surrounding tissues removed and kept in 10% buffered neutral formalin. The materials were processed by standard methods [44]. Paraffin blocks were made and sections were cut. These sections were stained with hematoxylin and eosin and mounted on glass slides. The histopathological changes were observed and recorded.

### 2.9. HPLC Quantitation

Different biological activities of HM have been reported in recent years [22,45,46], but no method for quantitation has been reported so far. It is important to standardize the different parts of HM, because of its widespread availability in various geographic regions and to detect its adulteration with other materials. Hence, the quantitation of HMF and HML was carried out by using two marker compounds, hyperoside and rutin. The extracts were dissolved in methanol (1 mg/mL) and filtered through Whatman filter paper, and the filtrate was used for HPLC analysis. Ten milligrams of standards hyperoside and rutin were dissolved separately in 5 mL of methanol in 10 mL volumetric flasks, and the volume was made up to 10 mL with the same solvent. Various concentrations were prepared from the stock solution and used for HPLC analysis. Chromatographic separation was performed on a Shimadzu^®^ liquid chromatographic system (Shimadzu, Kyoto, Japan), equipped with a LC-10AT-vp solvent delivery system, an Shimadzu^®^ SPD M-10AVP photo diode array detector (Shimadzu, Kyoto, Japan) and a Rheodyne 7725i injector (Sigma-Aldrich, St. Louis, MO, USA), with a 5-μL loop. A Phenomenex GEMINI C18 column (Phenomenex, Torrance, CA, USA) (25 cm × 4.6 mm i.d., 5 μm) was used for the separation. A mixture (75:25 v/v) of phosphate buffer (25 mM %) and acetonitrile was used as the mobile phase. It was delivered at a flow rate of 1.0 mL per min with detection at 360 nm. The retention time of hyperoside was found to be 5.80 min. The injection volume of the HMF and HML extracts was 50 μL. Analysis was performed at ambient temperature. Based on the peak area of standard and sample solution, the amount of hyperoside and rutin (%) was calculated.

### 2.10. Statistical Analysis

The experimental results were expressed as means ± S.E.M. The significance of the *in vivo* results was analyzed by one-way analysis of variance (ANOVA) followed by Tukey–Kramer multiple comparison tests, and *p* < 0.05 was considered as statistically significant.

## 3. Results and Discussion

### 3.1. Preparation of Extracts

The yield of dried methanolic extracts after Soxhlet extraction was 12.5% ± 0.25%, 10.33% ± 0.29%, 9.25% ± 0.11%, 4.21% ± 0.06% and 4.1% ± 0.02%, respectively for HMA, HMF, HML, HMR and HMS of HM. Phytochemical studies of these extracts showed the presence of saponins, flavonoids and tannins in all the parts of HM.

### 3.2. Estimation of Total Phenolic and Flavonoid Content

The total phenolic content and flavonoid content of the extracts were estimated by standard procedures [38,39]. The flowering top extract (HMF) possessed maximum total phenol and flavonol content with 38.6 and 33.48 mg/g of extract, respectively, followed by leaves, aerial parts, stem and root (Table 1).

**Table 1:** Total flavonol and phenol content of different extracts of *Hypericum mysorense* (HM).

Part of Plant	Total Flavonol Content * (mg/g of Extract)	Total Phenol Content * (mg/g of Extract)
HMF	33.48 ± 2.1	38.6 ± 3.66
HML	28.76 ± 1.44	37.07 ± 2.90
HMA	27.52 ± 1.67	32.7 ± 1.23
HMS	26.54 ± 3.56	30.7 ± 3.14
HMR	15.84 ± 0.84	20.7 ± 1.11

* The average of three determinations; values are the mean ± S.E.M. HMF, flowering tops; HML, leaves; HMA, aerial parts; HMS, stem; HMR, root.

### 3.3. In Vitro Antioxidant Activity

Results of the *in vitro* studies carried out for determining the antioxidant capacity of the methanolic extracts of different parts of HM in scavenging DPPH^•^, ABTS^•−^, NO^•^, O_2_^•−^ and ^•^OH radicals, model lipid and H_2_O_2_ are shown in Table 2. Positive correlations were observed between the results from total phenolic and total flavonoid contents (Table 1) with that of *in vitro* antioxidant activity using different assays (Table 2). HMF had the highest IC_50_ values, followed by HML, HMA, HMS and HMR, respectively. A comparison of the *in vitro* antioxidant efficiencies of the extracts with the standards, especially in the case of DPPH^•^, ^•^OH (*p*-NDA), O_2_^•−^, lipid peroxidation and total antioxidant capacity, showed that the HMF had better scavenging efficiency than the corresponding standards.

The ability of extracts to inhibit lipid peroxidation by inactivating free radicals was determined by preventing the oxidation of egg lecithin, using the TBA colorimetric method. The good efficiency of extracts rich in total phenols/flavonoids to inhibit lipid peroxidation may be due to the scavenging of free radicals and partly due to their ability to form stronger chelates with iron/copper ions, thus rendering them inactive towards undergoing the Fenton reaction in contrast to butylated hydroxyanisole and the α-tocopherol used as standards.

Many oxidase enzymes are known to generate H_2_O_2_ endogenously. Though by itself a weak oxidant, it can undergo a Fenton-type reaction to form potent ^•^OH radical; hence, it was of interest to study the scavenging of H_2_O_2_. The present studies showed that the extracts were relatively less efficient (by 1.5- to three-times) in scavenging H_2_O_2_ in comparison to rutin, used as a standard. The very high IC_50_ values in the ^•^OH radical scavenging by the well-known deoxyribose method, as well as by *p*-NDA methods showed that extracts, as well as the standards do not scavenge the radicals well. The reason for this phenomenon is rather obscure. The hydroxyl radical reacts with phenolic compounds present in the extracts by different mechanisms: firstly, by direct abstraction of hydrogen atom present in the ^•^OH group of the phenol, a typical example being the scavenging of DPPH^•^ radical; secondly, by the electron transfer process from the phenol to the free radical, followed by rapid proton transfer.

The *in vitro* ABTS^•−^, DPPH^•^, O_2_^•−^ radical scavenging methods allow one to determine, exclusively, the intrinsic ability of an antioxidant compound or of similar compounds present in extracts, to donate hydrogen atoms/electrons to these radicals in a homogenous system. The results obtained from these methods, though useful, however, cannot be directly extrapolated to biological systems, where the species are distributed in micro-heterogeneous systems according to their lipophilicities.

### 3.4. In Vivo Antioxidant and Hepatoprotective Studies

Among the five extracts tested, the HMF and HML showed potent *in vitro* antioxidant activity and total phenol and flavonol content. Hence, these two extracts were selected for *in vivo* antioxidant studies using the CCl_4_-intoxicated model using male albino rats of the Wister strain.

A significant increase in the levels of ASAT, ALAT, ALP and total bilirubin and a significant decrease in the levels of total proteins in liver and serum were observed in CCl_4_-intoxicated rats when compared to the normal control group. The HMF at 100 and 200 mg/kg, and HML at 200 mg/kg significantly (*p* < 0.001) restored the biochemical parameters towards normal. The HML at 100 mg/kg restored ASAT, ALAT and ALP levels significantly, but did not show significant restoration of total protein and bilirubin levels. HMF at 200 mg/kg and silymarin at 100 mg/kg showed potent restoration of all biochemical parameters towards a normal level (Table 3).

**Table 2:** *In vitro* antioxidant activity of methanol extracts of different parts of *Hypericum mysorense*. ABTS, diammonium salt; DPPH, 1,1-diphenyl-2-picrylhydrazyl; *p*-NDA, *p*-nitroso dimethyl aniline.

Extracts/Standards	IC_50_ Values ± S.E.M. (µg/mL) * by Methods								Total Antioxidant Capacity
	ABTS	DPPH	p-NDA	Hydrogen Peroxide	Lipid per Oxidation		Nitric Oxide	Alkaline DMSO	
HMF	1.53 ± 0.04 ^a,b^	3.65 ± 0.02 ^a,b^	690.15 ± 11.16 ^b,c^	57.56 ± 2.34 ^a,b^	13.68 ± 1.62 ^c,d^		208.35 ± 9.36	822.56 ± 18.93 ^a,b,c^	0.30 ± 0.01 ^d^
HML	2.05 ± 0.04 ^a,b^	4.05 ± 0.03 ^a,b^	>1000	68.34 ± 3.26 ^a,b^	18.47 ± 1.32 ^c,d^		250.53 ± 12.38	923.84 ± 22.45 ^a,b,c^	0.43 ± 0.01 ^d^
HMA	2.97 ± 0.04 ^a^	5.23 ± 0.04 ^a,b^	>1000	74.44 ± 3.32 ^a^	28.26 ± 1.76 ^c,d^		301.38 ± 12.57	>1000	0.66 ± 0.02 ^d^
HMS	3.63 ± 0.02 ^a^	5.95 ± 0.03	>1000	80.50 ± 4.02 ^a^	33.32 ± 1.34 ^c,d^		365.64 ± 15.64	>1000	0.96 ± 0.03 ^d^
HMR	4.60 ± 0.04 ^a^	8.32 ± 0.06	>1000	97.53 ± 2.98 ^a^	40.22 ± 2.13 ^c,d^		408.24 ± 14.75	>1000	1.43 ± 0.06 ^d^
**STANDARDS**									
Ascorbic acid	11.25 ± 0.49	2.69 ± 0.05	-	187.33 ± 3.93		-	-	>1000	-
Rutin	0.51± 0.01	3.91 ± 0.10	>1000	36.66 ± 0.22		-	65.44 ± 2.56	>1000	-
Butylated hydroxyl anisole	-	-	>1000	24.88 ± 0.16		112.66 ± 1.32	-	>1000	-
α-Tocopherol	-	-	-	-		91.66 ± 4.92	-	-	3.41 ± 0.47

* The average of three determinations; values are the mean ± S.E.M. For the deoxyribose method, the IC_50_ values of all the extracts were >1000 μg/mL. For total antioxidant capacity, the values are expressed as the equivalent of ascorbic acid per gram of extract. ^a^ *p* < 0.05, ^b^ *p* < 0.05, ^c^ *p* < 0.05, ^d^ *p* < 0.05 between extracts and standards of ascorbic acid, rutin, butylated hydroxyl anisole and α-tocopherol, respectively.

**Figure 1:**
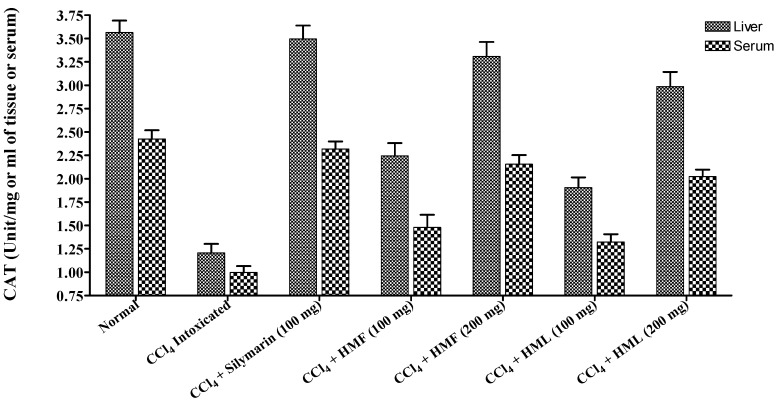
The effect of HMF and HML on CAT enzyme levels in liver and serum.

**Figure 2:**
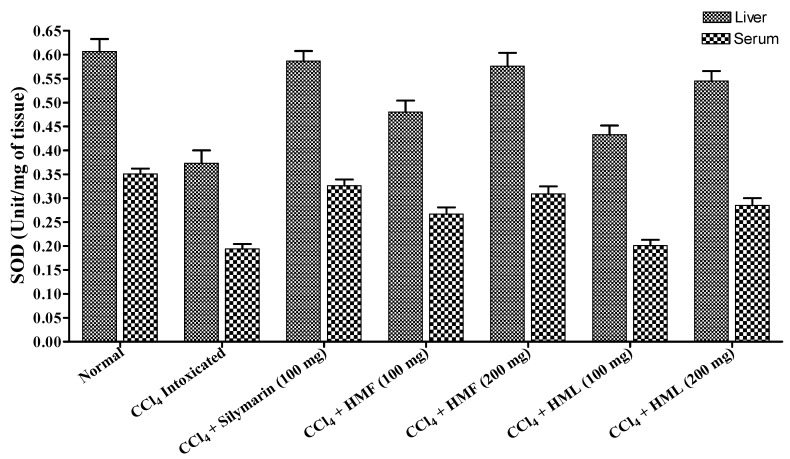
The effect of HMF and HML on SOD enzyme levels in liver and serum.

CCl_4_-intoxicated rats, showed a significant decrease in the levels of CAT and SOD and a significant increase in the TBARS levels when compared to the normal control rats. The HMF and HML at 200 mg/kg showed a significant (*p* < 0.001) reduction in the TBARS levels with an increase in SOD and CAT levels. The HMF and HML at 100 mg/kg showed moderate restoration of SOD, CAT and TBARS levels towards normal. HMF at 200 mg/kg showed potent restoration of enzymes and lipid peroxidation, comparable to that of standard silymarin (*p* < 0.001) (Figure 1 and Figure 2, Table 4).

**Table 3:** The effects of treatment with HMF and HML on the biochemical hepatoprotective parameters of CCl_4_-intoxicated rats. ASAT, aspartate amino transferase; ALAT, alanine amino transferase; ALP, alkaline phosphatase.

Treatment	Dose (mg or mL/Kg b.w.)	ASAT (U/L)		ALAT (U/L)		ALP (U/L)		Total Protein (G/dL)		Total Bilirubin (mg/dL)	
		Liver	Serum	Liver	Serum	Liver	Serum	Liver	Serum	Liver	Serum
**Normal**	-	101.52 ± 1.078	123.39 ± 1.244	51.02 ± 0.926	69.49 ± 1.435	197.83 ± 4.321	260.31 ± 6.622	6.917 ± 0.175	6.283 ± 0.207	0.516 ± 0.031	0.702 ± 0.036
**CCl_4_ intoxicated**	1 mL	390.74 ± 1.254 ^a^	450.23 ± 1.630 ^a^	198.57 ± 1.708 ^a^	237.58 ± 1.931 ^a^	515.23 ± 11.746 ^a^	691.05 ± 15.282 ^a^	4.867 ± 0.088 ^a^	4.692 ± 0.129 ^a^	1.783 ± 0.047 ^a^	2.017 ± 0.127 ^a^
**CCl_4_ + Silymarin**	1 mL + 100 mg	143.07 ± 1.633 ^b^	188.82 ± 1.493 ^b^	64.16 ± 1.071 ^b^	94.02 ± 1.317 ^b^	256.28 ± 9.387 ^b^	289.54 ± 7.784 ^b^	6.283 ± 0.105 ^b^	5.983 ± 0.194 ^b^	0.708 ± 0.063 ^b^	0.966 ± 0.042 ^b^
**CCl_4_ + HMF**	1 mL + 100 mg	292.75 ± 1.414 ^b^	304.62 ± 2.139 ^b^	160.34 ± 1.838 ^b^	180.47 ± 2.072 ^b^	428.52 ± 11.455 ^b^	547.53 ± 11.846 ^b^	5.556 ± 0.131 ^c^	4.933 ± 0.185	1.310 ± 0.068 ^b^	1.517 ± 0.047 ^b^
	1 mL + 200 mg	152.73 ± 1.062 ^b^	219.79 ± 1.282 ^b^	66.19 ± 1.052 ^b^	107.73 ± 1.606 ^b^	282.37 ± 10.147 ^b^	328.59 ± 9.316 ^b^	6.023 ± 0.116 ^b^	5.758 ± 0.180 ^c^	0.753 ± 0.062 ^b^	0.903 ± 0.036 ^b^
**CCl_4_ + HML**	1 mL + 100 mg	315.24 ± 1.695 ^b^	331.32 ± 2.274 ^b^	171.93 ± 1.517 ^b^	194.76 ± 1.942 ^b^	469.83 ± 14.269	584.38 ± 16.682 ^b^	5.032 ± 0.066	4.796 ± 0.169	1.483 ± 0.054 ^c^	1.717 ± 0.063 ^d^
	1 mL + 200 mg	187.03 ± 1.732 ^b^	254.42 ± 1.904 ^b^	76.69 ± 1.217 ^b^	121.59 ± 1.585 ^b^	307.27 ± 9.665 ^b^	361.31 ± 11.926 ^b^	5.767 ± 0.168 ^b^	5.413 ± 0.171	0.883 ± 0.043 ^b^	1.220 ± 0.066 ^b^

The results are the mean ± S.E.M. (*n* = 6), ^a^ *p* < 0.001, between the normal and CCl_4_-intoxicated groups. ^b^ *p* < 0.001, ^c^ *p* < 0.01, ^d^ *p* < 0.05, between the CCl_4_-intoxicated and treated groups.

**Table 4:** The effect of HMF, HML and silymarin on antioxidant enzymes and lipid peroxidation in CCl_4_-induced rats. TBARS, 2-thiobarbituric acid reactive substance; MDA, malondialdehyde.

Treatment	Dose (mg/kg Body Weight)	CAT (Unit/mg of Tissue)		SOD (Unit/mg of Tissue)		TBARS (*n* mole of MDA/mg of Protein)	
		Liver	Serum	Liver	Serum	Liver	Serum
**Normal**	0.5 mL Sodium CMC	3.564 ± 0.128	2.425 ± 0.094	0. 607 ± 0.026	0.351 ± 0.011	4.872 ± 0.178	3.845 ± 0.065
**Control (CCl_4_)**	1 mL	1.207 ± 0.096 ^a^	0.997 ± 0.069 ^a^	0.373 ± 0.027 ^c^	0.194 ± 0.010 ^a^	8.425 ± 0.149 ^a^	6.583 ± 0.176 ^a^
**Silymarin + CCl_4_**	100	3.495 ± 0.145 ^b^	2.318 ± 0.082 ^b^	0.587 ± 0.021 ^b^	0.326 ± 0.013 ^b^	5.102 ± 0.170 ^b^	4.207 ± 0.123 ^b^
**HMF + CCl_4_**	100	2.245 ± 0.137 ^d^	1.481 ± 0.133 ^b^	0.480 ± 0.024 ^c^	0.267 ± 0.014 ^d^	7.214 ± 0.243 ^b^	5.512 ± 0.114 ^b^
	200	3.310 ± 0.154 ^b^	2.156 ± 0.098 ^b^	0.576 ± 0.028 ^b^	0.309 ± 0.016 ^b^	5.456 ± 0.162 ^b^	4.417 ± 0.107 ^b^
**HML + CCl_4_**	100	1.906 ± 0.109	1.323 ± 0.084 ^d^	0.433 ± 0.019	0.201 ± 0.012	7.676 ± 0.196	5.878 ± 0.134 ^c^
	200	2.987 ± 0.156 ^b^	2.023 ± 0.075 ^b^	0.545 ± 0.021 ^d^	0.285 ± 0.015 ^b^	5.873 ± 0.112 ^b^	4.765 ± 0.124 ^b^

The results are the mean ± S.E.M. (*n* = 6), ^a^ *p* < 0.001, between the normal and CCl_4_-intoxicated groups. ^b^ *p* < 0.001, ^c^ *p* < 0.01, ^d^ *p* < 0.05, between the CCl_4_-intoxicated and treated groups.

Antioxidant action has been reported to play a crucial role in the hepatoprotective capacity of many plants, such as *Curcuma longa*, *Ganoderma formosanum*, *Solanum nigrum*, *Boehmeria nivea* and *Spirulina maxima* [47,48,49,50,51]. Thus, the search for drugs of plant origin with antioxidant activity has become a central focus of study of hepatoprotection. This may prove effective in alleviating tissue damage prevalent in organisms as a consequence of exposure to toxins of extrinsic or intrinsic origin. Natural antioxidants strengthen the endogenous antioxidant defenses, by ROS scavenging and restoring the optimal balance by neutralizing the reactive species. They are gaining immense importance by virtue of their critical role in disease prevention.

The preventive action against liver damage by CCl_4_ has been widely used as an indicator of the liver protective activity of drugs in general [52]. Since the changes associated with CCl_4_-induced liver damage are similar to that of acute viral hepatitis [53], CCl_4_-mediated hepatotoxicity was chosen as the experimental model. It has been established that CCl_4_ is accumulated in hepatic parenchyma cells and metabolically activated by cytochrome P450-dependent monooxygenases to form a trichloromethyl radical (CCl_3_). The CCl_3_ radical alkylates cellular proteins and other macromolecules with a simultaneous attack on polyunsaturated fatty acids, in the presence of oxygen, to produce lipid peroxides, leading to liver damage [54]. Thus, antioxidant or free radical generation inhibition is important in protection against CCl_4_-induced liver lesions [41]. ASAT, ALAT, ALP, TB and TP are the most sensitive tests for the diagnosis of liver diseases [55]. Hepatotoxic compounds, such as CCl_4_, are known to cause marked elevation in serum enzymes and bilirubin levels. It causes a marked decrease in TP levels. The present study revealed a significant increase in the activities of ASAT, ALAT, ALP and TB and a significant decrease in TP within 24 h of exposure to CCl_4_, in toxicant control animals, indicating considerable hepatocellular injury. Silymarin is used as a standard hepatoprotective compound, since it is reported to have a protective effect on the plasma membrane of hepatocytes [56]. Administration of HM extracts, especially HMF, attenuated the increased levels of the serum enzymes, produced by CCl_4_, and caused a subsequent recovery towards normalization, almost like that of the silymarin treatment, as reported [57].

Thus, the antioxidant activity or the inhibition of the generation of free radicals is important for the protection against CCl_4_-induced hepatopathy [41]. The body has an effective defense mechanism to prevent and neutralize the free radical-induced damage. This is accomplished by a set of endogenous antioxidant enzymes, such as SOD and CAT. These enzymes constitute a mutually-supportive team of defense against ROS [58]. In CCl_4_-induced hepatotoxicity, the balance between ROS production and these antioxidant defenses may be lost, and “oxidative stress” results, which, through a series of events, deregulates the cellular functions, leading to hepatic necrosis. The reduced activities observed for SOD and catalase point to the hepatic damage in the rats administered with CCl_4_ [59]. However, the extract-treated groups showed a significant increase in the level of these enzymes, which indicates the antioxidant potential of the HMF and HML.

The level of lipid peroxide is a measure of membrane damage and alterations in the structure and function of cellular membranes. In the present study, the elevation of lipid peroxidation in the liver of rats treated with CCl_4_ was observed. The increase in malondialdehyde (MDA) levels in liver suggests enhanced lipid peroxidation, leading to tissue damage and the failure of antioxidant defense mechanisms to prevent the formation of excessive free radicals [60]. Treatment with HMF and HML significantly reversed these changes. From the results, it is clear that the drugs show dose-dependent activity, among which, the HMF and HML at a dose level of 200 mg/kg p.o. showed greater activity, which is comparable with the standard drug, silymarin.

It is already been discussed that HMF and HML successfully restored the level of MDA after CCl_4_ intoxication towards normal. Both of the extracts prevented the lipid peroxidation in a dose-dependent manner, thereby preventing the hepatotoxicity, indicating the anti-lipid peroxidative effect. This suggested the possibility that HM extracts are able to condition the hepatocytes, so as to cause accelerated regeneration of parenchyma cells, thus protecting against membrane fragility and decreasing the leakage of the marker enzymes into circulation.

### 3.5. Histopathology

**Figure 3:**
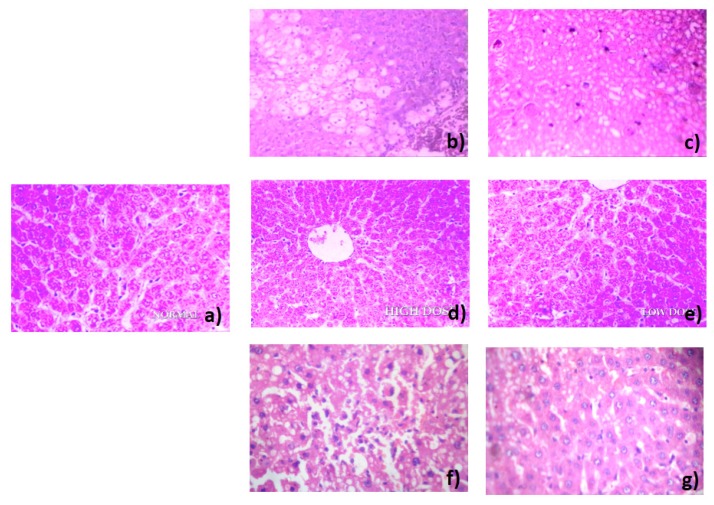
Histology of liver stained with hematoxylin-eosin (×100) of the groups of mice. (**a**) Normal; (**b**) CCl_4_-intoxicated; (**c**) CCl_4_ + silymarin treated; (**d**) CCl_4_ + HMF (200 mg/kg b.w.); (**e**) CCl_4_ + HMF (100 mg/kg b.w.); (**f**) CCl_4_ + HML (200 mg/kg b.w.); (**g**) CCl_4_ + HML (100 mg/kg b.w.).

Histological examination of the liver tissues under light microscope was done to observe the effects of HMF and HML on the structural integrity of the cells. The liver of normal animals showed a normal histological appearance (Figure 3a). The CCl_4_-intoxicated animal liver showed fat changes, liver necrosis and hepatic degeneration (Figure 3b). The animals treated with standard silymarin at the 100 mg/kg body weight dose and HMF at 200 and 100 mg/kg body weight doses exhibited an almost normal histological appearance of liver cells, except a few lymphocytic collections in the portal area (Figure 3c–e). The animals treated with HML at the 200 mg/kg body weight dose also showed normal histology with intact hepatocytes with slight inflammation (Figure 3f,g), whereas, 100 mg/kg body weight showed slightly more inflammatory changes compared to normal.

### 3.6. HPLC Quantitation

**Figure 4:**
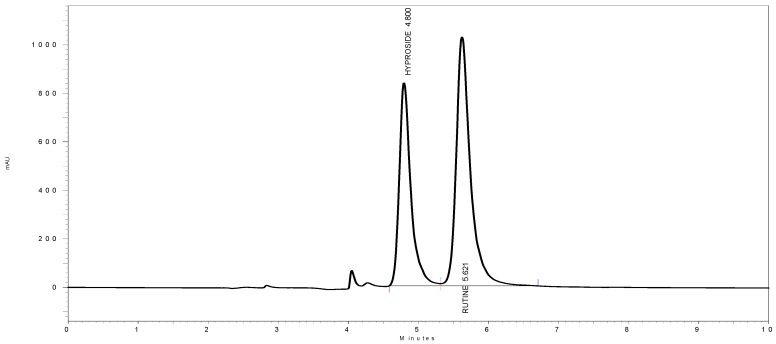
HPLC chromatographs of hyperoside and rutin.

**Figure 5:**
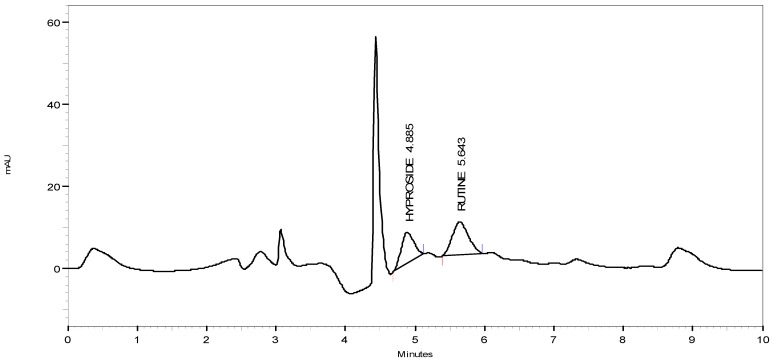
HPLC chromatographs of HMF.

**Figure 6:**
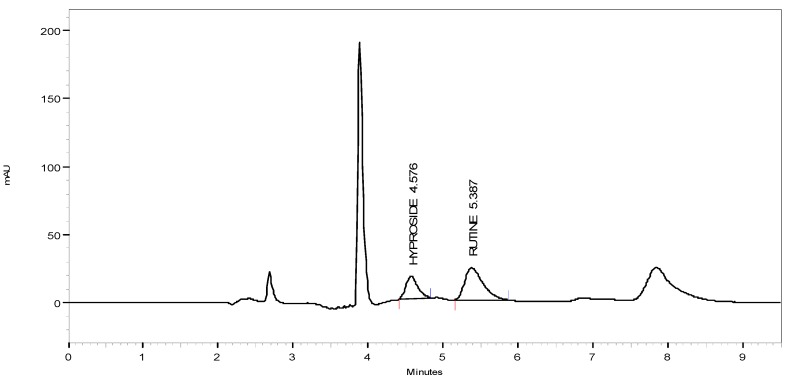
HPLC chromatographs of HML.

Typical chromatograms of hyperoside and rutin and of HMF and HML are shown in Figure 4, Figure 5 and Figure 6, respectively. The amount of hyperoside present in the HMF and HML was 1.981% ± 0.12% and 1.165% ± 0.09% w/w, respectively. The amount of rutin present in HMF and HML was 1.527% ± 0.1% and 1.238% ± 0.09% w/w, respectively.

## 4. Conclusions

The protective effect exhibited by HMF and HML against free radical-induced toxicity could be due to the protection of hepatic drug metabolizing enzymes and their antioxidant activities. The hepatic injury caused by CCl_4_ is associated with damage to the endoplasmic reticulum, and any compound capable of preventing the toxicity of CCl_4_ must have some direct or indirect effect on the liver. Both HMF and HML extracts have a maximum quantity of phenols and flavonols in them. The antioxidant activity of phenolics and flavonoids is well known and widely accepted. HMF and HML showed potent *in vitro* and *in vivo* antioxidant and hepatoprotective activity among the various extracts of HM. The antioxidant and hepatoprotective activity of HM may be due to its rich flavonoid content. Two compounds were isolated from the HMF and HML, namely, hyperoside and rutin.
